# Combined Therapeutics for Atherosclerosis Treatment Using Polymeric Nanovectors

**DOI:** 10.3390/pharmaceutics14020258

**Published:** 2022-01-22

**Authors:** Baltazar Hiram Leal, Brenda Velasco, Adriana Cambón, Alberto Pardo, Javier Fernandez-Vega, Lilia Arellano, Abeer Al-Modlej, Víctor X. Mosquera, Alberto Bouzas, Gerardo Prieto, Silvia Barbosa, Pablo Taboada

**Affiliations:** 1 Colloids and Polymers Physics Group, Department of Particle Physics, Faculty of Physics and Health Research Institute, Universidade de Santiago de Compostela, 15782 Santiago de Compostela, Spain; bahlemar@gmail.com (B.H.L.); brendavero.iq@gmail.com (B.V.); alberto.44444@gmail.com (A.P.); javier.fernandez.vega@rai.usc.es (J.F.-V.); liliagarellanog@gmail.com (L.A.); 2 Institute of Materials, Universidade de Santiago de Compostela, 15782 Santiago de Compostela, Spain; xerardo.prieto@usc.es; 3 Department of Physics and Astronomy, College of Science, King Saud University, Riyadh 11451, Saudi Arabia; amodlej@KSU.EDU.SA; 4 Cardiac Surgery Department, University Hospital of A Coruña, Biomedical Research Institute of A Coruña (INIBIC), 15006 A Coruña, Spain; Victor.x.mosquera.rodriguez@sergas.es (V.X.M.); alboumos@hotmail.com (A.B.); 5 Biophysics and Interfaces Group, Department of Applied Physics, Faculty of Physics, Universidade de Santiago de Compostela, 15782 Santiago de Compostela, Spain

**Keywords:** nanoparticles, combination therapy, atherosclerosis, miRNA, statin, inflammation

## Abstract

Atherosclerosis is an underlying risk factor in cardiovascular diseases (CVDs). The combination of drugs with microRNAs (miRNA) inside a single nanocarrier has emerged as a promising anti-atherosclerosis strategy to achieve the exploitation of their complementary mechanisms of action to achieve synergistic therapeutic effects while avoiding some of the drawbacks associated with current systemic statin therapies. We report the development of nanometer-sized polymeric PLGA nanoparticles (NPs) capable of simultaneously encapsulating and delivering miRNA-124a and the statin atorvastatin (ATOR). The polymeric NPs were functionalized with an antibody able to bind to the vascular adhesion molecule-1 (VCAM1) overexpressed in the inflamed arterial endothelium. The dual-loaded NPs were non-toxic to cells in a large range of concentrations, successfully attached overexpressed VCAM receptors and released the cargoes in a sustainable manner inside cells. The combination of both ATOR and miRNA drastically reduced the levels of proinflammatory cytokines such as IL-6 and TNF-α and of reactive oxygen species (ROS) in LPS-activated macrophages and vessel endothelial cells. In addition, dual-loaded NPs precluded the accumulation of low-density lipoproteins (LdL) inside macrophages as well as morphology changes to a greater extent than in single-loaded NPs. The reported findings validate the present NPs as suitable delivery vectors capable of simultaneously targeting inflamed cells in atherosclerosis and providing an efficient approach to combination nanomedicines.

## 1. Introduction

Cardiovascular diseases (CVDs) are the main cause of death worldwide, with 17.6 M deaths in 2019, which is projected to increase to 23.6 M by 2030 due to the increase in the population of elderly people and the average lifestyle of society, with atherosclerosis being the main underlying factor [1,2]. This is a complex chronic inflammatory and metabolic disease widely associated with genetic predisposition and multiple risk factors such as hypertension, hyperlipidemia, and diabetes mellitus [3]. Atherosclerosis is initiated by the dysfunction of the endothelial layer that can arise as early as adolescence and leads to the accumulation and retention of cholesterol-containing, low-density lipoproteins (LdLs) in the sub-endothelial space due to a complex interplay between activated leukocytes (monocytes, macrophages, and T cells) and the inflamed endothelium. This phenomenon progresses through the formation of atheromatous plaques in the intima of arteries, which often significantly compromise the residual lumen. This may lead to ischemic events and even the total occlusion of the artery or plaque rupture, which causes life-threatening artery diseases such as myocardial infarction (MI) or ischemic stroke (IS). In addition, atherosclerotic lesions do not form randomly; rather, they preferentially occur at vascular niches proximal to vessel branches and bends including several locations in the aortic arch, ascending and descending aorta, and coronary and carotid arteries [4]. At these locations, the local flow behavior is characterized as “disturbed” and is associated with low shear stress recirculation (average < 0.4 Pa) in comparison with straight regions of arteries with “normal” high shear stress, which show laminar pulsatile flow (average > 1 Pa; maximum < 10 Pa). Additionally, prolonged exposure to excessive cyclic circumferential stretch derived from heart beating resulted in the enhanced generation of vasoactive mediators, pro-inflammatory markers and matrix metalloproteinases [5,6,7,8], which also favor vessel endothelial inflammation and atherogenesis.

A major challenge in addressing atherosclerosis is that patients are generally asymptomatic until the very late stages of the disease, when significant vessel occlusion or a traumatic acute event caused by plaque rupture occurs. Early indicators of CVDs, such as non-natural levels of lipoproteins, glucose, blood pressure, and body weight, currently used to assess the disease and to recommend preventive systemic therapy to avoid future acute atherosclerotic-derived clinical events are only risk factors, not true and reliable diagnostic tools [9]. In fact, mechanisms involved in the different phases of atheroma formation and development are complex, but lipid metabolism is known to play a pivotal role [10,11]. In this regard, statin treatment, with its lipid-lowering effect and pleiotropic activity, is one of the main therapies of choice to fight against atherosclerosis in clinical practice through the systemic administration of the drug, but adverse side effects (mainly myopathy, liver damage, digestive system problems, or low blood platelet count) and low concentrations in the target atheroma represent major issues that must be addressed to improve clinical outcomes [12]. Hence, strategies that locally and specifically target the lipid component and the inflammatory burden of atheroma might overcome the present limitations.

Polymeric nanocarriers may be designed to offer inherent drug protection while in transit to the inflamed arterial endothelial tissue and can be easily surface-functionalized with specific moieties to interact with receptor endothelial targets with high affinity, allowing their efficient uptake by endothelial cells while locally releasing the encapsulated bioactive cargo without significant peripheral metabolism [13,14,15,16,17]. Hence, this approach may reduce the need for repeated drug administration and cause a reduction in peripheral and central toxicity, drug degradation, and off-target effects [18,19]. Amongst the different types of polymeric nanoparticles (NPs), those made of poly(lactic-co-glycolic acid), PLGA, are particularly appealing provided that this copolymer is biocompatible and biodegradable, since it undergoes hydrolysis in physiological medium to produce metabolite monomers, lactic acid, and glycolic acid. Moreover, this copolymer is commercially available in a wide range of compositions and molecular weights, which helps in regulating the cargo release profiles, as well as being approved by regulatory agencies to be used in several drug delivery systems as polymeric NPs as microspheres, injectable formulations, in situ forming and/or solid implants [20,21]. PLGA NPs exhibit a high stability and loading capacity of drugs and offer various feasible routes of administration, which is particularly interesting in the case of statins. PLGA NPs were selected in some previous prospective studies to construct new statin-loaded delivery systems for localized drug administration [22]. For example, pitavastatin-loaded PLGA NPs developed by Katsuki et al. were shown to inhibit monocyte recruitment via the under-expression of chemo-attractant proteins and their stimulating factors upon statin sustained release, thus reducing plaque destabilization and rupture [23]. These NPs also achieved a targeted drug release in an area of ischemia reperfusion injury, reducing the extension of myocardial infarction together with an improvement in ventricular function in a preclinical model of acute myocardial infarction [24]. In addition, such NP-based nanovehicles showed the enhanced protection of the myocardial extracellular matrix from post-ischemic remodeling events through the inhibition of monocyte mobilization from bone marrow [25]. Li et al. prepared RGD-modified PLGA NPs loaded with the glucocorticoid dexamethasone for active endothelial cell targeting to reduce the oxidative damage of human vein endothelial cells upon exposure to oxidized, low-density lipoproteins (ox-LdL). The controlled release of the drug upon application of external ultrasounds (US) allowed a decrease in oxidative stress and, hence, a much higher rate of cell survival in vitro [26]. In another study, Yokoyama et al. also used PLGA NPs to target simvastatin in mice ischemic myocardia [27]. The simvastatin-loaded PLGA NPs significantly enhanced cell migration and growth factor expression in vitro, and intravenous administration induced endogenous cardiac regeneration in their model of ischemic heart disease.

Despite all of these advances, the combination of statins with other bioactive compounds or biomolecules in a single polymeric nanocarrier to look for multimodal simultaneous therapies making use of complementary pathways to achieve enhanced synergistic therapeutic outcomes has hardly been explored. In particular, micro RNAs (miRNAs) are particularly appealing. miRNAs are small, non-coding, 22-nucleotide-long RNAs that bind complementary sequences of target messenger RNAs (mRNAs) to induce their degradation or repress translation [28]. The revelation that miRNAs function as potential oncogenes and tumor suppressors has generated great interest in using them as targets and/or therapeutic molecules for cancer therapy, and different nanovehicles co-encapsulating miRNA and drugs have been tested for multimodal cancer therapy by exploiting the different biochemical pathways to exert their respective therapeutic activities and obtained better therapeutic outcomes at lower doses [29,30,31,32]. However, this approach has been much less explored for other diseases and, in particular, for CVDs.

For this reason, in this work, we designed a polymeric nanocarrier based on PLGA NPs able to simultaneously encapsulate a statin drug and a gene-based material to provide a multimodal therapy against atherosclerosis. To achieve such a goal, the statin atorvastatin was solubilized in the core of the polymeric NPs through an emulsion process, whereas a miRNA-124a as genetic material was complexed on the NPs surfaces. The ability of the drug not only to lower the LdL levels in blood but also to provide pleiotropic effects such as the reduction in atherosclerotic plaque levels and anti-inflammatory activity can be exploited in combination with the demonstrated anti-inflammatory activity of the selected miRNA by means of complementary pathways [33,34,35]. Both the drug and the miRNA were successfully incorporated within the nanometric-sized NPs, which were colloidally stable in physiological-mimicking media. To ensure the localized and sustained release of the bioactive cargoes in the inflamed cells/tissues, active targeting was promoted by means of the functionalization of the polymeric NPs with an antibody (anti-CD106) able to bind with high affinity to the vascular adhesion molecule-1, VCAM1, receptor overexpressed in inflamed endothelial cells. It was observed that the NPs were efficiently taken up by both inflamed endothelial cells and macrophages, and that the combination of both the drug and the genetic material led to a more efficient reduction in inflammation, as observed from the reduced levels of inflammatory cytokine production as well as a decrease in ox-LdL internalization in activated macrophages and vascular endothelial cells.

## 2. Materials and Methods

### 2.1. Materials

Poly(d,l-lactide-co-glycolide) (PLGA) of molecular weight 24–38 kDa with a 50:50 lactide–glycolide ratio, poly(vinyl alcohol) (PVA), Pluronic F127, sodium periodate, bovine serum albumin, liposaccharides (LPS), oxidized low-density lipoprotein modified with the dye Dil, ox-LdL-Dil, and miRNA-124a ([5′–3′] sequence: UAAGGCACGCGGUGAAUGCC) were purchased from Sigma-Aldrich (St. Louis, MO, USA). Low-molecular-weight chitosan (LMW-chitosan, MW = 111 kDa) was purchased from Fluka (St. Louis, MO, USA). Atorvastatin with 95% purity was purchased from Alfa Aesar (Karlshrue, Germany). IL-6 and TNF-α ELISA kits (Thermo Fisher, Waltham, MA, USA), fluorescently labeled ICAM and VCAM antibodies, Dulbecco’s modified eagle medium (DMEM), fetal bovine serum (FBS), L-glutamine, penicillin/streptomycin, sodium pyruvate, and non-essential amino acids were purchased from Invitrogen (Carlsbad, CA, USA). A Qubit microRNA assay kit and ProLong Gold antifade reagent with DAPI were procured from Molecular Probes (Thermo Fisher, Waltham, MA, USA). Endothelial cell growth medium was procured from Cell Applications Inc., San Diego, CA, USA. Dialysis membrane tubing (molecular weight cutoff 3500) was purchased from Spectrum Laboratories, Inc. (Rancho Dominguez, CA, USA). A reactive oxygen species (ROS) analysis kit was procured from BQCkit (Oviedo, Spain). All other chemicals and solvents were of reagent grade (from Sigma-Aldrich, St. Louis, MO, USA). RNAse-free water was used in all preparations.

### 2.2. Synthesis of ATOR-Loaded PLGA/miRNA NPs

In a typical preparation, a 2.5 mL solution of PLGA in acetone (10 mg/mL) was mixed with 1 mL of ATOR solution at 10 mg/mL in methanol and gently mixed for 30 min. To this mixed solution, 50 mL of a Pluronic F127 aqueous solution (10 mg/mL) was added using an automatic syringe pump at a flow of 0.1 mL/min under stirring at 500 rpm at 10 °C. Next, the suspension was sonicated for 15 min at 100 W using a probe-type sonicator (20 kHz, Bandelin Sonopuls, Bandelin GmbH, Berlin, Germany) in an ice bath to homogenize the resulting dispersion. The organic solvent (acetone and ethanol) was completely evaporated under mechanical stirring overnight, and the dispersion was subsequently centrifuged twice at 9000 rpm for 30 min and 20 °C. Then, the supernatant was removed, and the final precipitate was resuspended in 5 mL of suitable solvent (water or PBS buffer when corresponding).

On the other hand, miRNA-124a was complexed to the ATOR-loaded PLGA NPs by electrostatic interactions with positively charged, low-molecular-weight chitosan (LMW-chitosan) present on the surface of the polymeric–PLGA NPs. Firstly, 5 mL of ATOR-loaded PLGA NPs suspension was mixed with 0.25 mL of 10 mg/mL LMW-chitosan previously dissolved in 50 mM acetic acid solution. After stirring for 4 h, the NPs were centrifuged twice at 9000 rpm for 30 min to eliminate free and loosely adsorbed chitosan. For the miRNA assembly, the desired amount of miRNA (typically 0.5 nmol) was diluted in 1 mL of water and stirred at 500 rpm for 30 min at 37 °C. Then, the ATOR-loaded PLGA NPs solution was added dropwise. After 2 h of incubation at 37 °C, the NPs were centrifuged twice at 9000 rpm for 30 min at 37 °C, redispersed in 5 mL of water or buffer solution, and stored at 4 °C for further use. Supernatants were recovered and used for the estimation of miRNA complexation. ATOR-loaded PLGA NPs were functionalized with VCAM1 antibody to analyze active targeting against inflamed endothelial cells. To ensure that antibodies were attached to the NP surfaces without compromising the recognition structural units, an oriented covalent strategy based on the oxidation of the sugar moieties of the antibody was chosen [36,37]. Hence, to oxidize the sugar residues (carbohydrates containing cis-diols groups) of the antibody molecules, NaIO4 was used as the oxidizing agent. As the negative control, a primary antibody (Ab) produced in mouse against lysosomal-associated membrane protein 1 was used (DSHB Cat# h4a3, RRID:AB_2296838), was obtained from the Developmental Studies Hybridoma Bank, created by the NICHD of the NIH and maintained at The University of Iowa, Department of Biology, Iowa City, IA 52242 (#CD107a). Both anti-VCAM and H4A3 Abs were initially quantified via a Coomassie protein assay. Once adjusted to the same protein concentration, an identical amount of Ab sample was added to NPs. A 97.5 mL amount of 0.2 M sodium periodate and 270 mL of antibody (ca. 1.8 mg/mL) were mixed (1500:1 molar ratio) in 135 mL of 10 mM PBS buffer, pH 7.4 for 2 h at 4 °C. The sample was then poured into a PD-10 molecular exclusion column (GE Healthcare #52-1308-00), collecting ca. 2 mL of Ab solution after the removal of the NaIO4 excess. A total of 10 mg of ATOR-loaded PLGA/miRNA NPs was incubated with 1 mL of a 10 mg/mL anti-VCAM solution in 10 mM PBS pH 7.4 for 2 h at 37 °C. Then, a solution of NaCNBH3 in 10 mM NaOH (375× excess regarding the antibody, 750 mL) was added for 30 min at 37 °C, followed by several washes with 10 mM MES buffer at pH 6.0 and subsequent incubation with 3 mL of a 10 mg/mL bovine serum albumin (BSA) solution to block non-specific binding sites. The conjugation extent was analyzed using a Comassie protein assay of the remaining supernatants as well as using a specific ELISA kit following the manufacturer’s instructions. Finally, the sample was washed three times (30 min, 800 rcf) and resuspended in 3 mL of 10 mM sodium phosphate buffer, pH 7.4.

### 2.3. NPs Characterization

NP sizes were obtained via dynamic light scattering (DLS) at 25 °C by means of an ALV-5000F (ALV-GmbH, Hessen, Germany) instrument with vertically polarized incident light (λ = 488 nm) supplied by a diode-pumped Nd:YAG solid-state laser (Coherent Inc., Santa Clara, CA, USA) operated at 2 W, and combined with an ALV SP-86 digital correlator (sampling time 25 ns to 100 ms). The experiment duration was in the range of 5–10 min, and each experiment was repeated at least three times. The sizes and morphologies of the NPs were also acquired via transmission and scanning electron microscopy (TEM and SEM, respectively) by means of Phillips Zeiss Libra 200 Fm Omega and Zeiss FESEM Ultra Plus electronic microscopes (Oberkochen, Germany) operating at 120 and 20 kV, respectively. The NPs’ zeta potential was measured in triplicate with a Zetasizer Nano ZS (Malvern Panalytical, Malvern, UK), using disposable folded capillary cells. The UV–Vis and fluorescence spectroscopy spectra of the particles were performed in Cary 100 Bio and Cary Eclipse spectrophotometers (Agilent Technologies, Santa Clara, CA, USA), respectively. The concentration of PLGA NPs was determined using a nanoparticle tracking analysis (NTA) instrument (NanoSight, Malvern Panalytical, Malvern, UK).

### 2.4. Cargo Entrapment Efficiency and Loading Capacity

To quantify the amount of miRNA complexed to the NPs, the concentration of the remaining miRNA in the supernatant after complexation was estimated by means of a Qubit microRNA assay kit following the manufacturer´s instructions. On the other hand, the encapsulation of ATOR inside the PLGA NPs was estimated from the direct measurement of the encapsulated drug after NPs’ dissolution in dichloromethane assisted by sonication as well as by analyzing the supernatants resulting from the NP washing steps along the encapsulation process. Drug solutions were analyzed by means of high-performance liquid chromatography (HPLC) using a Jasco chromatograph (series LC-400) equipped with a PU-1480 pump, UV–Vis detector, oven (model CO-4061) and autosampler (model AS-1450), and a C-18 column (250 × 4.6 mm, 5 μm pore size), Jasco International Co., Tokio, Japan. Measurements were made in isocratic mode by injecting 5 μL of the sample using acetonitrile-water (55:45%) as the mobile phase at a flux of 1.0 mL/min and registration at a wavelength of 212 nm. ATOR concentration was calculated from a calibration curve of free drug at well-known concentrations by calculating the area under the chromatogram curve after a retention time of ca. 3 min.

Drug loaded/conjugated, *D.L.*, and entrapment efficiency, *E.E.*, in the ATOR–loaded PLGA/miRNA NPs were calculated as follows:

(1) $$D.L.\%=\frac{weight\;of\;ATOR;miRNA\;in\;NPs}{weight\;of\;NPs}\times 100$$

(2) $$E.E.\%=\frac{weight\;of\;ATOR;miRNA\;in\;NPs}{initial\;weight\;of\;ATOR;miRNA}\times 100$$

### 2.5. In Vitro Release Kinetics

Aliquots (1 mL) of ATOR-loaded PLGA and ATOR-loaded PLGA/miRNA NPs in PBS pH 7.4 and citrate buffer at pH 5.0 were placed in a temperature-controlled bath at 37 °C with a stirring speed of 100 rpm. The samples were collected at regular time intervals and measured via HPLC (ATOR) or using a Qubit microRNA assay kit as previously described.

### 2.6. Cell Culture

Mouse 264.7 RAW macrophages and human vein endothelial (HUVEC) cell lines were purchased from Cell Biolabs (San Diego, CA, USA). RAW cells were cultured at standard conditions (37 °C, 5% CO_2_) in Dulbecco’s Modified Eagle Medium, DMEM, supplemented with 10% (*v*/*v*) FBS and 1% (*v*/*v*) penicillin/streptomycin, sodium pyruvate, and nonessential amino acids (NEAAs). HUVEC cells were cultured in full endothelial cell growth medium.

### 2.7. In Vitro Cytotoxicity

The cytotoxicity of ATOR-loaded PLGA/miRNA NPs was evaluated by means of the CCK-8 proliferation assay (Dojindo Molecular Technologies, Rockville, MD, USA). Cells were seeded into 96-well plates (100 μL, 1.0 × 10^4^ cells per well) and incubated for 24 h at standard culture conditions (5% CO_2_ at 37 °C) in their respective culture medium. Afterwards, ATOR-loaded PLGA/miRNA NPs and the corresponding controls (free ATOR, ATOR-loaded PLGA NPs, and PLGA/miRNA NPs) were injected into the plate wells (100 μL) at different particle concentrations (5 × 10^9^–1.0 × 10^11^ NP/mL) and incubated for 24 h. After incubation, the culture medium was discarded, and the cells were washed with cold 10 mM PBS, pH 7.4 several times. Next, 100 μL of culture medium containing 10 μL of CCK-8 reagent was added and left for incubation for 1 h. The *Cell cytotoxicity* was quantified via UV–Vis at 450 nm using a microplate reader as follows:

(3) $$\%\;Cell\;cytotoxicity=\frac{Abs\;sample}{Abs\;blank}\times 100$$

where *Abs sample* is the absorbance at 450 nm for cells incubated with NPs, and *Abs blank* is the absorbance for controls without NPs. Measurements were made at least in triplicate.

### 2.8. Cellular Uptake

For fluorescence microscopy analysis, mouse RAW 264.7 macrophage cells were seeded on poly-l-lysine-coated glass coverslips (12 × 12 mm^2^) placed inside 6-well plates (3 mL, 1 × 10^5^ cells per well) and grown for 24 h at standard culture conditions. Then, 300 µL of ATOR-loaded PLGA/miRNA NPs (50 mg/mL) was added to cells and incubated for 6 h. As a proof of concept, a fluorescent scramble miRNA sequence was used to test the ability of NPs to transport and release their cargo inside cells. After incubation, the NP-containing cells were washed three times with ice-cold PBS at pH 7.4, fixed with paraformaldehyde 4% (*w*/*v*) for 10 min, washed with PBS again, permeabilized with 0.2% (*w*/*v*) Triton X-100, washed with PBS twice, and stained with BODIPY Phalloidin (Invitrogen, Thermo Fisher, Waltham, MA, USA) for 30 min. Cells were washed again with cold PBS, mounted on glass slides, stained with ProLong Gold antifade DAPI (Invitrogen, Thermo Fisher, Waltham, MA, USA), and cured for 24 h at −20 °C. Samples were visualized with a 63× objective using a epifluorescence microscope Leica DMI6000B (Leica Microsystems GmbH, Heidelberg Mannheim, Germany), where the blue channel corresponds to DAPI (λex = 355 nm), the red channel to BODIPY Phalloidin (λex = 633 nm), and the transmitted light was observed in differential interference contrast (DIC) mode. The green channel corresponds to the FITC dye labeling of the scramble miRNA sequence.

### 2.9. Cellular Morphological Changes

The modification of the morphology of mouse RAW 264.7 macrophages after liposaccharide (LPS) stimulation and the inhibitory effect provided by ATOR-loaded PLGA/miRNA NPs was analyzed by seeding this type of cell on poly-l-lysine-coated glass coverslips (12 × 12 mm^2^) placed inside 6-well plates (3 mL, 1 × 10^5^ cells per well) and grown for 24 h at standard culture conditions. Then, 300 µL of ATOR-loaded PLGA/miRNA NPs (50 mg/mL) was added to cells and incubated for 24 h. After incubation, cells were treated with 100 ng/mL LPS and incubated for an additional 72 h. Afterwards, treated and control cells were washed with cold 10 mM PBS pH 7.4 three times, fixed with paraformaldehyde 4% (*w*/*v*) for 10 min, washed with PBS again, permeabilized with 0.2% (*w*/*v*) Triton X-100, washed with PBS twice, and stained with BODIPY Phalloidin (Invitrogen, Thermo Fisher, Waltham, MA, USA) for 30 min. Cells were washed again with cold PBS, mounted on glass slides, stained with ProLong Gold antifade DAPI (Invitrogen, Thermo Fisher, Waltham, MA, USA), cured for 24 h at −20 °C, and observed by fluorescence microscopy as described above.

### 2.10. Expression of Pro-Inflammatory Cytokines

The anti-inflammatory capacity of ATOR-loaded PLGA/miRNA NPs was analyzed by measuring the inhibition of inflammatory cytokines IL-6 and TNF-α upon exposure of mouse RAW 264.7 macrophages to LPS. Macrophages were seeded in 96-well plates at a density of 10,000 cells/well. After 24 h of incubation, cells were treated with 100 ng/mL LPS for 24 h; next, cells were washed with cold 10 mM PBS pH 7.4 twice, and 100 μL of a 50 mg/mL ATOR-loaded PLGA/miRNA NPs solution was added to the cells and incubated for a further 48 and 72 h. After incubation, the cell medium of each well was collected and analyzed to quantify the amount of pro-inflammatory cytokines IL-6 and TNF-α by means of corresponding ELISA kits following manufacturers’ instructions using a Fluorostar Omega plate reader (BMG Labtech, Ortenberg, Germany). Measurements were made at least in quintuplicate.

### 2.11. Quantification of Reactive Oxygen Species (ROS) Production

The inhibition of ROS production upon the stimulation of macrophages with LPS after the addition of ATOR-loaded PLGA/miRNA NPs was analyzed using a ROS assay kit based on the fluorogenic dye molecule 5(6)-Carboxy-2′,7′-dichlorofluorescein diacetate (DFCA-DA) from Abcam, Cambridge, UK. Cells were seeded in 96-well plates for 24 h. After incubation, a group of cells was treated with 100 ng/mL LPS for 24 h and were then washed with cold 10 mM PBS pH 7.4 twice, and 100 mL of a 50 mg/mL ATOR-loaded PLGA/miRNA NPs solution was added to the cells and incubated for a further 24 h. Afterwards, the ROS production was analyzed using the corresponding kit following the manufacturer’s instructions using a Fluorostar Omega plate reader (BMG Labtech, Ortenberg, Germany). Measurements were made at least in quintuplicate.

### 2.12. Oxidized, Low-Density Lipoproteins (Ox-LdL) Internalization

The potential decrease in ox-LdL uptake in activated mouse RAW 264.7 macrophages and HUVEC cells after treatment with ATOR-loaded PLGA/miRNA NPs was evaluated by means of fluorescence microscopy and flow cytometry. For microscopy, cells were seeded on poly-l-lysine-coated glass coverslips (12 × 12 mm^2^) placed inside 6-well plates (3 mL, 1 × 10^5^ cells per well) and grown for 24 h at standard culture conditions. After incubation, cells were treated with 100 ng/mL LPS and incubated for an additional 24 h. Then, 300 µL of ATOR-loaded PLGA/miRNA NPs (50 mg/mL) were added to cells and incubated for 24 h in cell culture medium deficient of FBS. Next, cells were washed with cold 10 mM PBS pH 7.4 twice, and culture medium containing ox-LdL (20 µg/mL) modified with the dye Dil ((2Z)-2-[(E)-3-(3,3-dimethyl-1-octadecylindol-1-ium-2-yl)prop-2-enylidene]-3,3-dimethyl-1-octadecylindole, perchlorate), Dil-ox-LdL, was added to cells and incubated for 4 h. Afterwards, cells were washed three times with PBS, fixed with paraformaldehyde 4% (*w*/*v*) for 10 min, washed with PBS another three times, permeabilized with 0.2% (*w*/*v*) Triton X-100, washed with PBS twice, and stained with BODIPY Phalloidin for 30 min. Cells were washed again with cold PBS, mounted on glass slides, stained with ProLong Gold antifade DAPI, cured for 24 h at −20 °C and observed using fluorescence microscopy as described previously.

For flow cytometry experiments, cells were seeded in 6-well plates (3 mL, 5 × 10^5^ cells/well) with the corresponding cell culture medium for 24 h. After incubation, cells were treated with 100 ng/mL LPS and incubated for an additional 24 h, and then, 1.5 mL of ATOR-loaded PLGA/miRNA NPs (50 mg/mL) was added to cells and incubated for 24 h in cell culture medium deficient of FBS. Next, cells were washed with cold 10 mM PBS pH 7.4 twice and culture medium containing Dil-ox-LdL (20 µg/mL) was added to cells and incubated for 4 h. Afterwards, cells were washed again three times with PBS to remove excess of Dil-ox-LdL and trypsinized with 0.25× trypsin for 4–8 min. Next, cells were centrifuged at 1200 rpm for 4 min, supernatants were discarded, and the cell pellets were washed with PBS three times to eliminate potential traces of trypsin. Cells were resuspended in 200 µL of PBS and analyzed in a Guava EasyCyte flow cytometer (Millipore, Burlington, MA, USA).

### 2.13. Evaluation of VCAM and ICAM-1 Expression

The enhancement in VCAM and ICAM-1 expression the after stimulation of HUVEC cells with LPS (100 µg/mL), and subsequent inhibition after treatment with ATOR-loaded PLGA/miRNA NPs was analyzed using fluorescence microscopy and flow cytometry. For microscopy, cells were seeded on poly-l-lysine-coated glass coverslips (12 × 12 mm^2^) placed inside 6-well plates (3 mL, 1 × 10^5^ cells per well) and grown for 24 h at standard culture conditions. After incubation, cells were treated with 100 ng/mL LPS and incubated for an additional 24 h. Then, 300 µL of ATOR-loaded PLGA/miRNA NPs (50 mg/mL) was added to cells and incubated for 24 h in cell culture medium deficient of FBS. Next, cells were washed with cold 10 mM PBS pH 7.4 twice, and new medium was added. Next, 5 µg/mL anti-CD106-PE (anti-VCAM) and anti-CD54-FITC (anti-ICAM-1) antibodies were added to cells and incubated for 4 h in the dark. Next, 2 mg/mL bovine serum albumin (BSA) was added to block non-specific binding sites. Cells were washed three times with PBS and fixed with paraformaldehyde 4% (*w*/*v*) for 10 min, washed with PBS three times, permeabilized with 0.2% (*w*/*v*) Triton X-100, washed again twice and mounted on glass slides, stained with ProLong Gold antifade DAPI, cured for 24 h at −20 °C, and observed using fluorescence microscopy as described previously.

For flow cytometry, HUVEC cells were seeded in 6-well plates (3 mL, 5 × 10^5^ cells/well) with the corresponding cell culture medium for 24 h. After incubation, cells were treated with 100 ng/mL LPS and incubated for an additional 24 h, and then, 1.5 mL of ATOR-loaded PLGA/miRNA NPs (50 mg/mL) was added to cells and incubated for 24 h in cell culture medium deficient of FBS. Next, cells were washed with cold 10 mM PBS pH 7.4 twice and culture medium containing 10 µg/mL anti-CD106-PE (anti-VCAM) and anti-CD54-FITC (anti-ICAM-1) antibodies were added to cells and incubated for 4 h in the dark. Next, 2 mg/mL bovine serum albumin (BSA) was added to block non-specific binding sites. Cells were washed three times with PBS and trypsinized with 0.25× trypsin for 4 min. Next, cells were centrifuged at 1200 rpm for 4 min, supernatants were discarded, and the cell pellets were washed with PBS three times to eliminate potential traces of trypsin. Cells were resuspended in 200 µL of PBS and analyzed in a Guava EasyCyte flow cytometer.

### 2.14. Statistical Analysis

Statistical evaluations of data were performed with Origin software (OriginLab Corporation, Northampton, MA, USA). All results were presented as mean standard errors unless otherwise indicated. One-way ANOVA (* *p* < 0.05, ** *p* < 0.01, *** *p* < 0.001) was used to determine statistical differences for multiple groups, whereas an unpaired *t*-test was used to analyze individual groups.

## 3. Results and Discussion

### 3.1. Synthesis and Characterization of ATOR-Loaded PLGA/miRNA NPs

Statins have been widely used as inhibitors of hydroxymethylglutharyl-coenzyme A reductase (HMG-CoA), a crucial enzyme in the early stage of the biosynthesis of cholesterol, to reduce the lipidic levels in blood. In recent years, this type of drug has been attributed to bear immunomodulatory and anti-inflammatory effects and is being currently used in the treatment of immunological and cardiovascular diseases such as rheumatoid arthritis, multiple sclerosis, and atherosclerosis [38,39,40]. On the other hand, miRNA-124a is produced in the cytoplasm, where the precursor miRNA hairpin is cleaved by endoribonuclease Dicer, forming the miRNA duplex. In particular, miRNA-124a, highly induced by α7 nicotinic acetylcholine receptor (α7nAChR) activation, mediates the cholinergic anti-inflammatory action by inhibiting the LPS-induced production of pro-inflammatory IL-6 cytokine and cytokine precursor genes, with this being one of the key actors together with TNF-α in inflammatory signaling [35]. Hence, the controlled administration of such bioactive compounds and the possibility to combine them in a single nanovehicle to achieve their protection and simultaneous sustained release opens new avenues for a multimodal therapeutic strategy for atherosclerotic treatment by making use of the different pathways these molecules exploit to exert their bioactivity.

**Figure 1:**
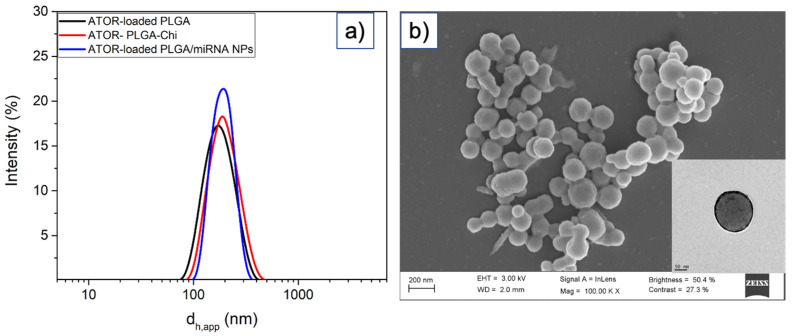
Hydrodynamic size populations (apparent hydrodynamic diameter, d_h,app_, of (**a**) ATOR-loaded PLGA (black line); ATOR-loaded PLGA stabilized with chitosan (red line), and ATOR-loaded PLGA/miRNA NPs (blue line). (**b**) SEM image of ATOR-loaded PLGA/miRNA NPs. The inset in (**b**) denotes a TEM image of the same NPs.

ATOR-loaded PLGA NPs were obtained by means of a single emulsion synthetic process. The optimization of the physico-chemical properties of the PLGA core matrix was previously carried out [41]. Next, miRNA was attached to the NPs by means of electrostatic interactions established with the positively charged, chitosan-coated PLGA NP surfaces. ATOR-loaded PLGA NPs exhibit a hydrodynamic size of ca. 164 ± 12 nm, which increases up to 196 ± 14 nm when miRNA is bound (Figure 1a,b), and have NP surface charges of +18.8 ± 4.1 and +4.9 ± 2.6 mV, respectively. The observed decrease in ζ-potential upon the complexation of miRNA-124a confirms the successful attachment of the genetic material onto the polymeric NPs. Nevertheless, the NP stability is not compromised due to the remaining electric charge as well the presence of steric polymeric hindrance, which preclude their aggregation. Additionally, the NP population size distributions appear well monodisperse, indicating that the particles are stable in solution with no signs of aggregation. The NPs display a spherical morphology as revealed by TEM images, with sizes rather similar to those derived from DLS data (ca. 186 ± 13 for ATOR-loaded PLGA/miRNA NPs as an example, see Figure 1). Finally, ATOR-loaded PLGA/miRNA NPs were functionalized with the VCAM antibody by means of an oriented covalent strategy, as described in the Section 2, to ensure the NPs targeted the endothelial inflamed cells. The extent of functionalization was of ca. four antibody molecules per NP, as detected using a Comassie blue assay and ELISA, and the NPs experienced only slight increases in size up to ca. 208 ± 23 bearing similar ζ- potentials of 5.9 ± 2.9 compared to non-functionalized NPs.

The successful incorporation of ATOR inside the hybrid NPs and the complexation extent of miRNA were corroborated using HPLC and fluorescence spectroscopy, respectively, as indicated in the Section 2. ATOR encapsulation efficiency and drug loading of ca. 89 ± 12% and 2.2 ± 0.4% were achieved after optimization of the initial fed drug concentration, that is, different initial drug concentrations were tested to achieve an optimal correlation between fed and encapsulated drug concentrations, which was slightly larger than most of the previously reported values for PLGA NPs, with values from ca. 65 up to 95% [41,42,43]; in the case of miRNA, an encapsulation efficiency of ca. 68% was determined using the Qubit RNA assay, similar to other miRNA encapsulated in PLGA NPs [44].

**Figure 2:**
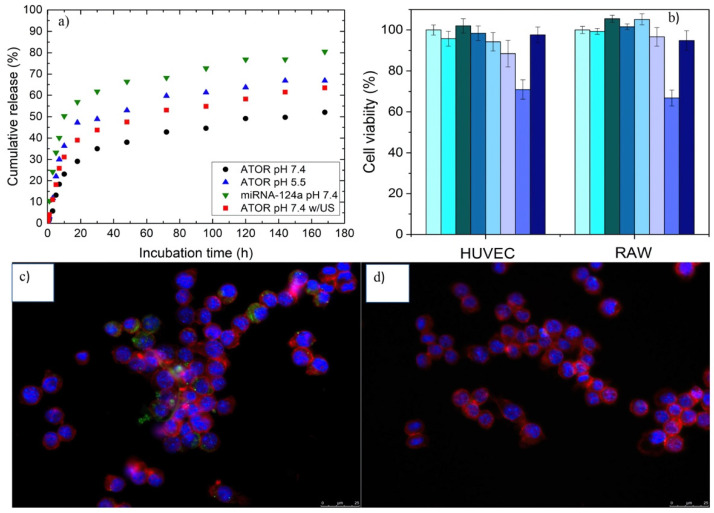
(**a**) Release profiles of ATOR at (●) pH 7.4, (■) pH 5.5, (▲) miRNA at pH 7.4, and (▼) ATOR release at pH 7.4 in the presence of applied ultrasounds from NPs. Error bars are not shown for clarity (uncertainties < 10%, three different replicates); (**b**) cell viability of HUVEC and RAW cells in the presence of polymeric NPs after 24 h of incubation. (From left to right) control LPS-activated cells, 5.0 × 10^9^, 1.0 × 10^10^, 2.5 × 10^10^, 5.0 × 10^10^, and 1.0 × 10^11^ NPs/mL of ATOR-loaded PLGA/miRNA NPs, free ATOR, and free miRNA. Internalization of (**c**) fluorescently labeled, ATOR-loaded PLGA/scramble miRNA NPs (5 × 10^10^ NPs/mL), and (**d**) free, fluorescently labeled, scramble miRNA in RAW 264.7 macrophages. Scale is 25 mm.

ATOR and miRNA-124a release profiles were determined using dialysis experiments under different conditions (Figure 2a). Additionally, ATOR release was analyzed in the absence and presence of applied US (1 W, 2 MHz, 5 min). In vitro cumulative ATOR release profiles at both neutral and acidic conditions in the presence of 10% (*v*/*v*) FBS showed a burst followed by a much slower diffusion-initiated release pattern (Figure 2a). In total, 23 and 31% ATOR was released from the NPs during the first 10 h of incubation, and 52 and 64% was released at 170 h for pH 7.4 and 5.5, respectively. ATOR release was additionally enhanced upon exposure of the drug-loaded polymeric NPs to US, achieving 50 and 81% after 10 and 170 h of incubation, respectively. This result confirmed that the present hybrid NPs can be controlled by the application of US to enhance the drug release on demand. This enhancement can be a consequence of the continuous expansion and contraction of the particle matrix promoted by the injected perfluorocarbon gas. This, in turn, also favors the acceleration of particle erosion [45] and the cargo escape from the NP core by an out-diffusion process through the core–shell structure, whose diffusion rate depends on factors such as copolymer crystallinity, viscosity, and drug association state [46]. Finally, the release of miRNA from the NPs at pH 7.4 was observed to be larger than that of the drug, with 36 and 67% after 10 and 170 h, respectively. This enhanced release rate compared to ATOR clearly stems from the location of the miRNA onto the NP surfaces; although miRNA is associated through complexation with chitosan, which stabilizes NPs, the Donnan effect and the presence of anionic species in solution help in progressively dissociating such complexes, thus favoring the release of the bioactive compound [47,48]. The observed incomplete cumulative release profiles of both cargoes result from the combination of a relatively limited time interval for release studies and the existence of strong polymer–cargo interactions, hindering the drug release [49,50].

### 3.2. Cytocompatibility and Cell Uptake ATOR-Loaded PLGA/miRNA NPs

The cell toxicities of activated (with LPS) RAW 264.7 macrophages and HUVEC cells treated with ATOR-loaded PLGA/miRNA NPs at different particle concentrations after 24 h of incubation were negligible, as observed in Figure 2b, with viabilities above 70% in all cases, thus confirming that these NPs can be considered as biocompatible. In fact, their toxicity is lower than that of free ATOR, probably due to a reduced drug concentration inside cells thanks to its sustained release from the NPs. In addition, the NPs were internalized to good extents, as detected by the observed green fluorescence of a fluorescently labeled scramble miRNA in the macrophage cytoplasm (Figure 2c). This fact confirms the incorporation of the NPs inside cells since when the labeled scramble miRNA is directly administered to cells, no fluorescence is observed provided that the genetic material is degraded in the biological milieu previous to the uptake (Figure 2d).

### 3.3. Therapeutic Activity of ATOR-Loaded PLGA/miRNA NPs

**Figure 3:**
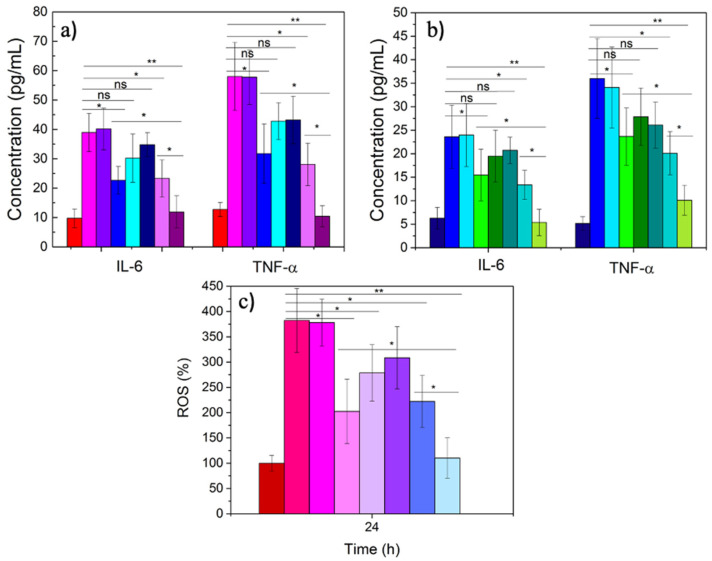
Production of cytokines in the absence and presence of the platform in (**a**) RAW 264.7 macrophages and (**b**) HUVEC cells; (**c**) ROS production in the absence and presence of the platform in RAW 264.7 macrophages. From left to right column: control cells, activated cells (100 ng/mL LPS), free miRNA (100 nM), free ATOR (20 mM), ATOR-PLGA NPs (5 × 10^10^ NPs/mL, 20 mM drug), PLGA/miRNA NPs (5 × 10^10^ NPs/mL, 100 nM miRNA), and ATOR-PLGA/miRNA NPs at 1.0 × 10^10^ (4 mM drug-20 nM miRNA) and 5.0 × 10^10^ NPs/mL (20 mM drug-100 nM miRNA). Statistical significance compared to LPS-activated cells: * = *p* < 0.05; ** = *p* < 0.01; ns = non statitiscallr significant.

As mentioned previously, IL-6 and TNF-α are two of the interleukins involved in the regulation of the inflammatory response at acute phase after, for example, an infection, and are usually used as biomarkers for such purposes [51]. They play a key role in affecting and controlling the behavior of different cells, such as progenitor myeloid, T and B cells, lymphocytes, and hepatocytes. Hence, we evaluated the potential combined anti-inflammatory effect of the developed dual ATOR-PLGA/miRNA NPs through the down-regulation effect of the miRNA on cytokine expression and the chemical action of the drug via ELISA assay. After the simultaneous stimulation of both RAW and HUVEC cells with LPS (100 ng/mL) and incubation with the NPs for 24 h, a large decrease in interleukin production was observed. Figure 3a shows that the combination of the miRNA and the drug is more effective than that of the single therapeutic cargoes alone, with a reduction from ca. 23 and 31 pg/mL to ca. 12 and 9 pg/mL corresponding to IL-6 and TNF-α in RAW cells for ATOR-loaded PLGA and ATOR-PLGA/miRNA NPs, respectively; hence, in the dual-therapeutic polymeric NPs, similar cytokine expressions to those of non-activated control macrophages are restored. Similar behavior was also noted for HUVEC cells (Figure 3b). It is also observed that the therapeutic effectiveness of the NPs is larger as the particle concentration increases within the range analyzed. Hence, the combination of both therapeutic cargoes within a single NP provides a combined inhibitory effect rather superior to the single encapsulation of each component and, of course, compared to the free administration of the molecules. The sustained and parallel release of both active compounds and their different and complementary pathways of action may then provide synergistic effects leading to the decrease in the analyzed inflammation biomarkers. The present results are in accordance with some previous studies. For example, Fei et al. administered simvastatin to BV2 microglial cells and noted that statin was involved in an important reduction in inflammatory cytokines (IL-6, IL-12, TNF-α, iNOS, etc) and the suppression of Notch signaling but also increased the levels of anti-inflammatory biomarkers such as IL-10, Arg-1, and CD206 [52].

The decrease in cytokine production can be directly related to the change in the polarization state of macrophages from a polarized inflammatory M1 state to an anti-inflammatory M2 one. This is also reflected in the ROS production in inflamed RAW macrophages upon activation with LPS in the absence and presence of therapeutic nanovehicles provided that this type of cell is the main contributor to this kind of inflammatory response. It has been shown that statins diminish ROS production in vitro and in vivo [53]. Figure 3c effectively shows that ROS production is largely inhibited in the presence of the dual-loaded, ATOR-loaded PLGA/miRNA NPs up to levels similar to the negative control (non-activated macrophages), and this inhibition becomes larger as the nanocarrier concentration increases. Additionally, it is noted that the dual-loaded NPs show a larger inhibitory effect than the single-loaded ones or upon free administered ATOR, corroborating the complementary role of both bioactive cargoes, which is additionally favored by the protection offered by the nanocarrier and the achieved sustained release.

**Figure 4:**
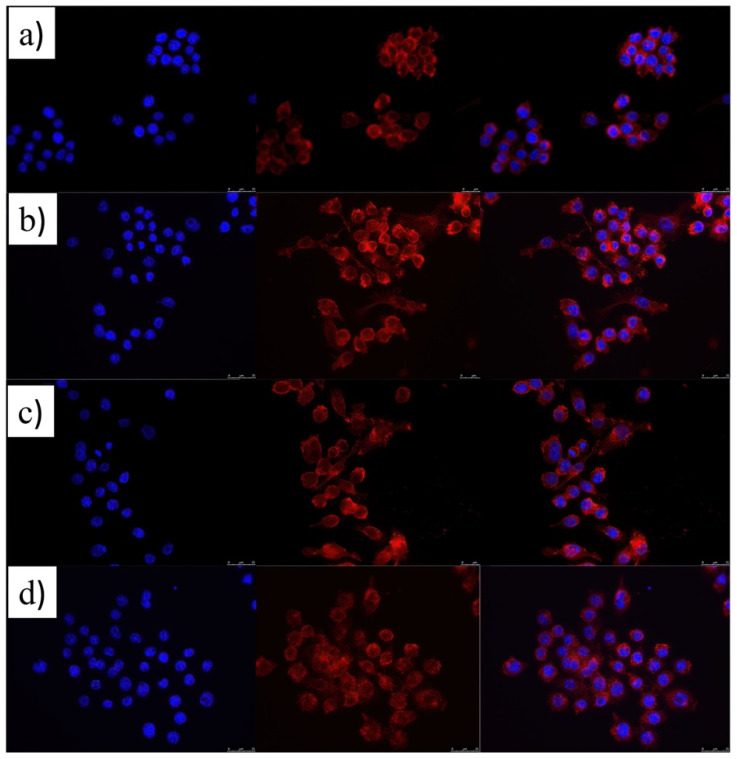
Morphology of RAW 264.7 macrophages: (**a**) non-activated; (**b**) activated with 100 ng/mL LPS; (**c**) after administration of 5 × 10^10^ NPs/mL of ATOR-PLGA NPs (20 mM drug); (**d**) after administration of 5 × 10^10^ NPs/mL ATOR-PLGA/miRNA NPs (20 mM drug–100 nM miRNA). From left to right: DAPI channel; BODIPY-Alexa Fluor 647 channel; merge image. Scale is 25 mm.

The modification in macrophage polarization state, which is reflected in the levels of production of cytokines and ROS, LdL trafficking, etc., can be also accompanied by changes in cell morphology. Figure 4a corroborates this point. It can be observed that non-activated (or M2) macrophages showed their typical spherical morphological particularly clearly when observing the staining of the cell cytoplasms.

When RAW cells are stimulated with LPS, typical branching in cell cytoskeletons giving rise to more elongated cell morphologies can be observed, in particular, from the red channel of the epifluorescence images in Figure 4b, which are characteristic of the inflamed M1 after cytokine release; nevertheless, the quasi-spherical cell morphology is recovered after the administration of the dual-loaded nanocarrier thanks to the combined therapeutic effect (Figure 4d), indicative of the restoration of the anti-inflammatory M2 polarization state supported by the important decrease in cytokines’ and ROS production after treatment, and which influence the decrease in macrophage and LdL trafficking and the subsequent accumulation at endothelial tissue [54,55]. However, this restoration is not fully achieved when ATOR-loaded PLGA NPs are injected to cells, since ca. 40–60% of RAW cells still showed morphological characteristics compatible with an inflamed polarization state (Figure 4c).

**Figure 5:**
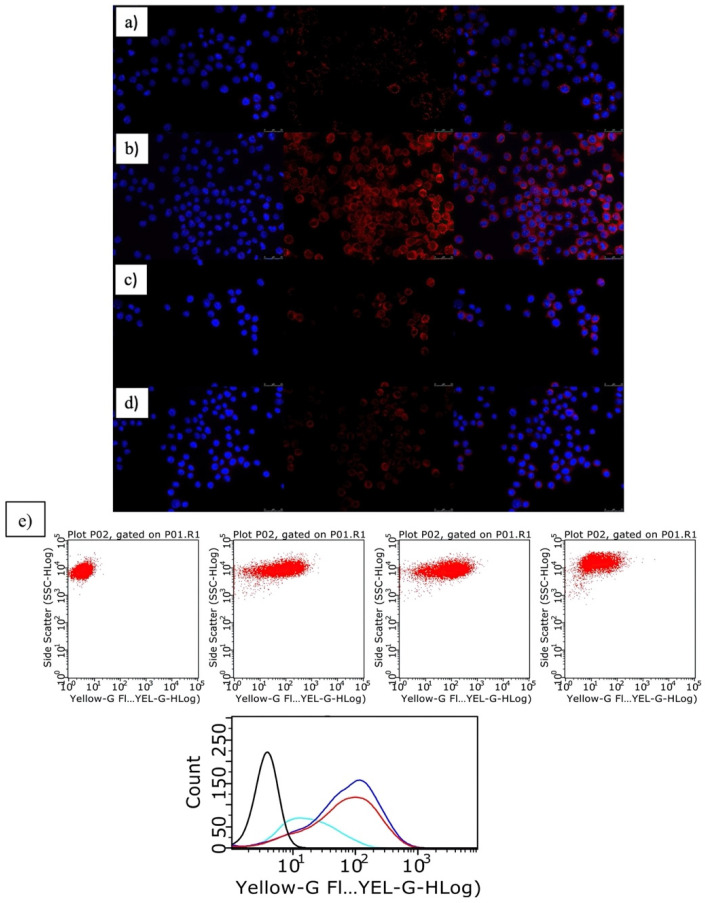
Ox-LdL by RAW 264.7 macrophages: (**a**) non-activated; (**b**) activated with 100 ng/mL LPS; (**c**) after administration of 1 × 10^10^ NPs/mL ATOR-PLGA NPs (20 mM drug); (**d**) after administration of 5 × 10^10^ NPs/mL ATOR-PLGA/miRNA NPs (20 mM drug–100 nM miRNA). From left to right: DAPI channel; Dil-ox-LdL channel; merge image. Scale is 25 mm. (**e**) Flow cytometry data of Dil-ox-LdL uptake in RAW cells: From left to right: bare, non-activated cells, bare, LPS-activated cells, and LPS-activated cells treated with 1.0 × 10^10^ and 5.0 × 10^10^ NPs/mL; bottom: fluorescence distribution of Dil-ox-LdL inside cells: (—) control, non-activated cells; (—) control, LPS-activated cells; LPS-activated cells treated with (—) 1.0 × 10^10^ and (—) 5.0 × 10^10^ NPs/mL.

In addition, another key factor in athero-progression is the maturation of the adhered macrophages in the endothelial tissue, which leads to the capture of LdL and modified LdL as oxidized-LdL (ox-LdL). The retrieval of ox-LDL takes place through scavenging receptors and contributes to the formation of the LdL-loaded macrophages, that is, to the so-called foam cells. Since the inhibition of inflammation and macrophage polarization is key for the formation of foam cells, we evaluated the efficacy of the present ATOR-loaded PLGA/miRNA NPs in diminishing or even preventing the internalization of ox-LdL in activated RAW cells. Figure 5a shows that non-inflamed cells hardly uptake the lipid molecules compared to the activated ones, for which an extensive cytoplasmatic internalization is observed (Figure 5b), as observed from the red fluorescence corresponding to the fluorescently labeled ox-LdL, in agreement with other studies [56]. 

Upon the administration of single ATOR-loaded PLGA NPs, a decrease in the red fluorescence signal from fluorescently labeled ox-LdL can be observed (Figure 5c), which is even more severe when the ATOR-loaded PLGA/miRNA NPs are incubated with the inflamed RAW cells (Figure 5d), in agreement with the data shown above. These observations from fluorescent microscopy images were corroborated using flow cytometry experiments (Figure 5e). Figure 5d confirms that the administration of ATOR-loaded PLGA/miRNA NPs is more effective than ATOR-loaded PLGA NPs in avoiding extensive ox-LdL uptake by activated macrophages, in which the role played by miRNA-124a in reducing IL-6 gene expression clearly helps in the reduction in inflammation and, thus, in ox-LdL trafficking and uptake.

Finally, the activity of ATOR-loaded PLGA/miRNA NPs in decreasing the overexpression of adhesion molecules, which play an important role in the recruitment of monocytes/macrophages, T cells and other types of leukocytes to the inflamed endothelial tissue [57], was tested. Among the different surface receptors in endothelial cells, adhesion molecules belonging to the family of integrins ICAM-I (CD54) and VCAM (CD106) have been demonstrated to be largely overexpressed in a time- and concentration-dependent manner [58] under several inflammation stimuli such as the presence of inflammatory cytokines such as TNF-α or IFN-γ and the accumulation of LdL in the arterial tissue [59,60].

**Figure 6:**
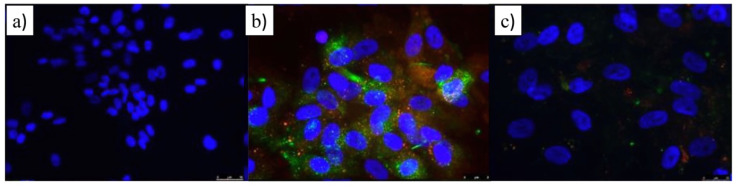
Fluorescent microscopy images denoting the expression of ICAM (green) and VCAM (orange–red) in (**a**) non-activated and (**b**) LPS-activated HUVEC cells, and (**c**) LPS-activated HUVECs after treatment with 5 × 10^10^ NPs/mL of ATOR-PLGA/miRNA NPs (20 mM drug–100 nM miRNA). In blue, DAPI channel; in red, labeled VCAM Ab; in green, labeled ICAM Ab. Scale is 25 mm.

Hence, HUVEC cells were stimulated with TNF-α, and the potential inhibitory effect of ATOR-loaded PLGA/miRNA NPs on the expression of ICAM-I and VCAM receptors was evaluated using immunostaining microscopy and flow cytometry. Figure 6 shows that non-activated HUVEC cells hardly showed a fluorescent signal, confirming the low expression of such endothelial receptors under normal physiological conditions. Conversely, upon stimulation with TNF-α, green and orange–red signals are observed, corresponding to the signals of CD54 and CD106 surface membrane staining, respectively, after fluorescently labeled antibody binding to their respective receptors. Here, it is necessary to note that the expression levels of ICAM-I are larger than VCAM as observed for the more extended green fluorescence. After the administration of ATOR-loaded PLGA/miRNA NPs, a decrease in the fluorescent signals of both receptors is clearly observed, indicative of the reduction in the expression of these inflammation biomarkers, and in agreement with the inhibition of interleukin production and decrease in ox-LdL accumulation described above promoted by the synergistic anti-inflammatory effects provided by the statin and miRNA-124-a through the mechanisms commented on previously.

## 4. Conclusions

miRNAs simultaneously regulate several inflammation-related genes, and their combination with statins opens the door to afford the treatment of inflamed endothelial vascular tissue, making use of complementary pathways to achieve synergistic therapeutic outcomes. To achieve such a goal, both bioactive compounds were simultaneously encapsulated in different compartments of PLGA NPs. In this manner, a protection to the sensitive genetic material and the statin drug is provided while achieving large encapsulation efficiencies and a sustained release pattern while maintaining the colloidally stable nanocarrier. Additionally, the release pattern can be externally controlled through the application of applied US energy, which would enable an on-demand dosage for the bioactive cargoes, and which will be the focus of a forthcoming publication. In addition, the dual-loaded nanocarrier was non-toxic to cells in a vast range of concentrations, allowing outstanding therapeutic effects in terms of decreased cytokine, ROS production, and ox-LdL trafficking and internalization in LPS-activated macrophages and HUVEC cells compared to free administered therapeutic or single cargo-loaded PLGA NPs, even at lower concentrations. In addition, the functionalization of the nanocarrier with an anti-VCAM antibody confirms the efficiency of a targeted delivery strategy to inflamed endothelial cells in vitro. The fabrication of this nanoconstruct is now being optimized for large-scale production under GMP conditions and tested in vivo in an atherosclerotic rat model, which will be the subject of a forthcoming publication.

## Data Availability

All data available are reported in the article.
